# Application value of the automated machine learning model based on modified CT index combined with serological indices in the early prediction of lung cancer

**DOI:** 10.3389/fpubh.2024.1368217

**Published:** 2024-04-05

**Authors:** Leyuan Meng, Ping Zhu, Kaijian Xia

**Affiliations:** ^1^Department of Respiratory and Critical Care Medicine, Affiliated Hospital of Nantong University, Medical School of Nantong University, Jiangsu, Nantong, China; ^2^Department of Scientific Research, The Changshu Affiliated Hospital of Soochow University, Jiangsu, Suzhou, China; ^3^Changshu Key Laboratory of Medical Artificial Intelligence and Big Data, Jiangsu, Suzhou, China

**Keywords:** automated machine learning, predictive models, infiltrative lung cancer, medical image artificial intelligence recognition system (MIARS), 7-TAABs

## Abstract

**Background and objective:**

Accurately predicting the extent of lung tumor infiltration is crucial for improving patient survival and cure rates. This study aims to evaluate the application value of an improved CT index combined with serum biomarkers, obtained through an artificial intelligence recognition system analyzing CT features of pulmonary nodules, in early prediction of lung cancer infiltration using machine learning models.

**Patients and methods:**

A retrospective analysis was conducted on clinical data of 803 patients hospitalized for lung cancer treatment from January 2020 to December 2023 at two hospitals: Hospital 1 (Affiliated Changshu Hospital of Soochow University) and Hospital 2 (Nantong Eighth People’s Hospital). Data from Hospital 1 were used for internal training, while data from Hospital 2 were used for external validation. Five algorithms, including traditional logistic regression (LR) and machine learning techniques (generalized linear models [GLM], random forest [RF], gradient boosting machine [GBM], deep neural network [DL], and naive Bayes [NB]), were employed to construct models predicting early lung cancer infiltration and were analyzed. The models were comprehensively evaluated through receiver operating characteristic curve (AUC) analysis based on LR, calibration curves, decision curve analysis (DCA), as well as global and individual interpretative analyses using variable feature importance and SHapley additive explanations (SHAP) plots.

**Results:**

A total of 560 patients were used for model development in the training dataset, while a dataset comprising 243 patients was used for external validation. The GBM model exhibited the best performance among the five algorithms, with AUCs of 0.931 and 0.99 in the validation and test sets, respectively, and accuracies of 0.857 and 0.955 in the validation and test groups, respectively, outperforming other models. Additionally, the study found that nodule diameter and average CT value were the most significant features for predicting lung cancer infiltration using machine learning models.

**Conclusion:**

The GBM model established in this study can effectively predict the risk of infiltration in early-stage lung cancer patients, thereby improving the accuracy of lung cancer screening and facilitating timely intervention for infiltrative lung cancer patients by clinicians, leading to early diagnosis and treatment of lung cancer, and ultimately reducing lung cancer-related mortality.

## 1 Introduction

Lung cancer is globally recognized as one of the malignancies with the highest incidence and mortality rates. According to the 2022 global cancer statistics survey, an average of approximately 350 individuals die from lung cancer every day, surpassing the combined total of breast, prostate, and pancreatic cancers. In China, lung cancer deaths account for 23.8% of the total cancer-related deaths, with the incidence and mortality rates ranking highest globally (1). Due to factors such as existing medical conditions and awareness of check-ups, many patients are diagnosed with late-stage lung cancer during their initial medical visits. Effective treatment options for late-stage lung cancer are limited, with a 5-year cumulative survival rate of only 19% (2). Early screening significantly improves the prognosis and survival of lung cancer patients (3), so early screening and diagnosis is the key to reduce lung cancer mortality and improve survival rate.

Currently, there is a lack of effective early screening methods, with emphasis placed on low-dose spiral computed tomography (LDCT) scans, biological tumor markers, and tumor autoantibody screening (4). However, these methods suffer from drawbacks such as high false positive rates, inadequate sensitivity, and suboptimal accuracy. Therefore, we attempt to accurately predict tumor malignancy and infiltration depth using an improved CT index obtained through artificial intelligence recognition technology combined with serum biomarkers consisting of lung cancer autoantibodies and tumor markers. This approach aims to assist clinicians in making more informed treatment decisions and improving patient survival benefits.

Machine learning, as a subset of artificial intelligence, has shown remarkable prospects in various fields such as economics, finance, business management, and bioinformatics. In the healthcare sector, it demonstrates outstanding applications in analyzing disease-related factors, predicting risks, and computer-aided diagnosis (5–7). Automated machine learning (AutoML) automates the application of machine learning to data by iteratively transforming data, selecting machine learning algorithms, and optimizing hyperparameters to choose the best model.

The aim of this study is to evaluate the predictive value of an improved CT index combined with serum biomarkers using a GBM model for early diagnosis of lung cancer. Clinical data from lung cancer patients from two hospitals were collected, and training, validation, and testing were conducted using the H2OAutoML platform. The performance of the GBM model was compared with traditional logistic regression (LR) to assess its efficacy.

## 2 Materials and methods

### 2.1 Inclusion and exclusion criteria

We retrospectively collected and analyzed data from patients who underwent lung cancer surgery at the Affiliated Changshu Hospital of Soochow University and Nantong Eighth People’s Hospital from January 2020 to December 2023. Patients collected from January 2020 to December 2023 at the Affiliated Changshu Hospital of Soochow University were used as the training set, while patients collected from October 2022 to December 2023 at Nantong Eighth People’s Hospital were used as the testing set.

The diagnostic criteria for lung cancer were referenced from the 2021 Fifth Edition of the WHO Classification of Thoracic Tumors (8). Diagnosis of lung cancer required meeting the following criteria: (1) Confirmation of lung nodules by chest CT without any clinical or drug intervention; (2) Definitive pathological results confirming benign or malignant nodules after chest CT; (3) Age ≥ 18 years; (4) Preoperative testing for 7 lung cancer autoantibodies and tumor markers; (5) Absence of significant dysfunction in other major organs; (6) Absence of other primary malignant tumors; and (7) Lung nodule diameter ≤ 3 cm. Exclusion criteria included: absence of pathological examination despite confirmed lung nodules on chest CT; failure to undergo testing for the 7 lung cancer autoantibodies and tumor markers; clinical or drug intervention prior to blood sampling; presence of rheumatic immunological diseases; lung metastasis from other tumors; lung nodule diameter > 3 cm. This study was approved by the hospital ethics committee.

### 2.2 Data collection

Demographic features, clinical information, and comorbidities were extracted from electronic medical records. Chest plain scans were performed using a 64-slice spiral CT scanner to obtain conventional CT imaging features, including air bronchogram sign, spiculated sign, lobulation sign, vascular penetration, pleural retraction, bronchial inflation sign, nodule diameter, and solid proportion. And the patient’s CT data were imported into the DeepRay medical image AI recognition system, which extracted quantitative features from medical images in high throughput and combined with convolutional neural networks to train deep learning on the data of the nodule’s size, density, and the proportion of solidity to get the improved CT indexes: the pulmonary nodule’s malignancy probability value and average CT value. Serum biomarkers primarily included 7 tumor-associated autoantibodies (TAABs) and commonly used tumor markers recommended by the American Clinical Biochemistry Committee and the European Tumor Marker Expert Group. TAABs detection involved extracting fasting peripheral venous blood (9–12) from patients preoperatively or before surgery. After centrifugation to separate serum, the levels of 7 lung cancer autoantibodies were measured using enzyme-linked immunosorbent assay (ELISA) (13), including tumor suppressor gene P53 (normal reference range: P53 < 13.09 U/mL), protein gene product PGP 9.5 (normal reference range: PGP9.5 < 11.1 U/mL), SRY-box containing gene 2 (normal reference range: SOX2 < 10.26 U/mL), G antigen 7 (GAGE7) (normal reference range: GAGE7 < 14.36 U/mL), RNA helicase autoantibody 4–5 (GBU4-5) (normal reference range: GBU4-5 < 6.99 U/mL), melanoma antigen A1 (MAGEA1) (normal reference range: MAGEA1 < 11.92 U/mL), and tumor-associated gene CAGE (normal reference range: CAGE <7.23 U/mL). TAABs detection results were considered positive if any of the indicators exceeded the normal reference range. Tumor markers were collected from blood tests and included primary lung cancer markers such as vascular endothelial growth factor (VEGF), carcinoembryonic antigen (CEA), neuron-specific enolase (NSE), cytokeratin fragment 19 (CYFRA21-1), pro-gastrin-releasing peptide (ProGRP), and squamous cell carcinoma antigen (SCC) (14).

### 2.3 Automated machine learning

Through the AI platform^1^, the H2O package is installed in the R language to implement AutoML analysis. Autonomy and automation are achieved through three aspects: feature selection, model construction, and hyperparameter optimization. The integrated algorithms include Generalized Linear Models (GLM), Random Forests (RF), Gradient Boosting Machines (GBM), Deep Neural Networks (DL), and Naive Bayes (NB), among others. The training set is split into development and validation sets in a 6:4 ratio, and blind verification is conducted with the testing set to evaluate the average accuracy and stability of the models. A confusion matrix consisting of true positives (TP), true negatives (TN), false positives (FP), and false negatives (FN) is established (15). Performance metrics including sensitivity, specificity, positive predictive value (PPV), negative predictive value (NPV), positive likelihood ratio (LR+), negative likelihood ratio (LR-), accuracy, area under the receiver operating characteristic curve (AUC), and the F1-Measure are calculated. Formulas for calculation are as follows: Accuracy = (TP + TN)/(TP + FP + FN + TN); PPV = TP/(TP + NP); NPV = TN/(TN + FN); LR + =Sensitivity/(1−Specificity); LR− = (1−Sensitivity)/Specificity; F1-Measure = (2*precisionrecall)/(precision+recall). Through SHAP analysis (Shapley Additive Explanations), an additive explanatory model is constructed to determine significant factors influencing model predictions and their contributions to model performance.

### 2.4 Statistical analysis

For continuous data, the Shapiro–Wilk test and homogeneity of variance test (Homogeneity of variance test) were first performed. For normally distributed and homoscedastic continuous data, independent samples t-tests were employed, and results were presented as mean ± standard deviation. For non-normally distributed and heteroscedastic continuous data, the Wilcoxon rank-sum test was used, and results were presented as median (M25, M75). Categorical data were expressed as frequencies and percentages, and inter-group differences were assessed using the chi-square test or Fisher’s exact test. To prevent multicollinearity among variables, feature selection was conducted using the Least Absolute Shrinkage and Selection Operator (LASSO) regression model. Based on the selected variables, a binary logistic regression model was fitted. The predictive performance of the obtained model was evaluated using the area under the receiver operating characteristic curve (AUC), calibration curve, and decision curve analysis (DCA), and a Nomogram was constructed. The statistical significance level was set at *p* < 0.05. All statistical analyses were performed using R 4.3.3 software.

## 3 Results

### 3.1 Baseline characteristics

**Figure 1:**
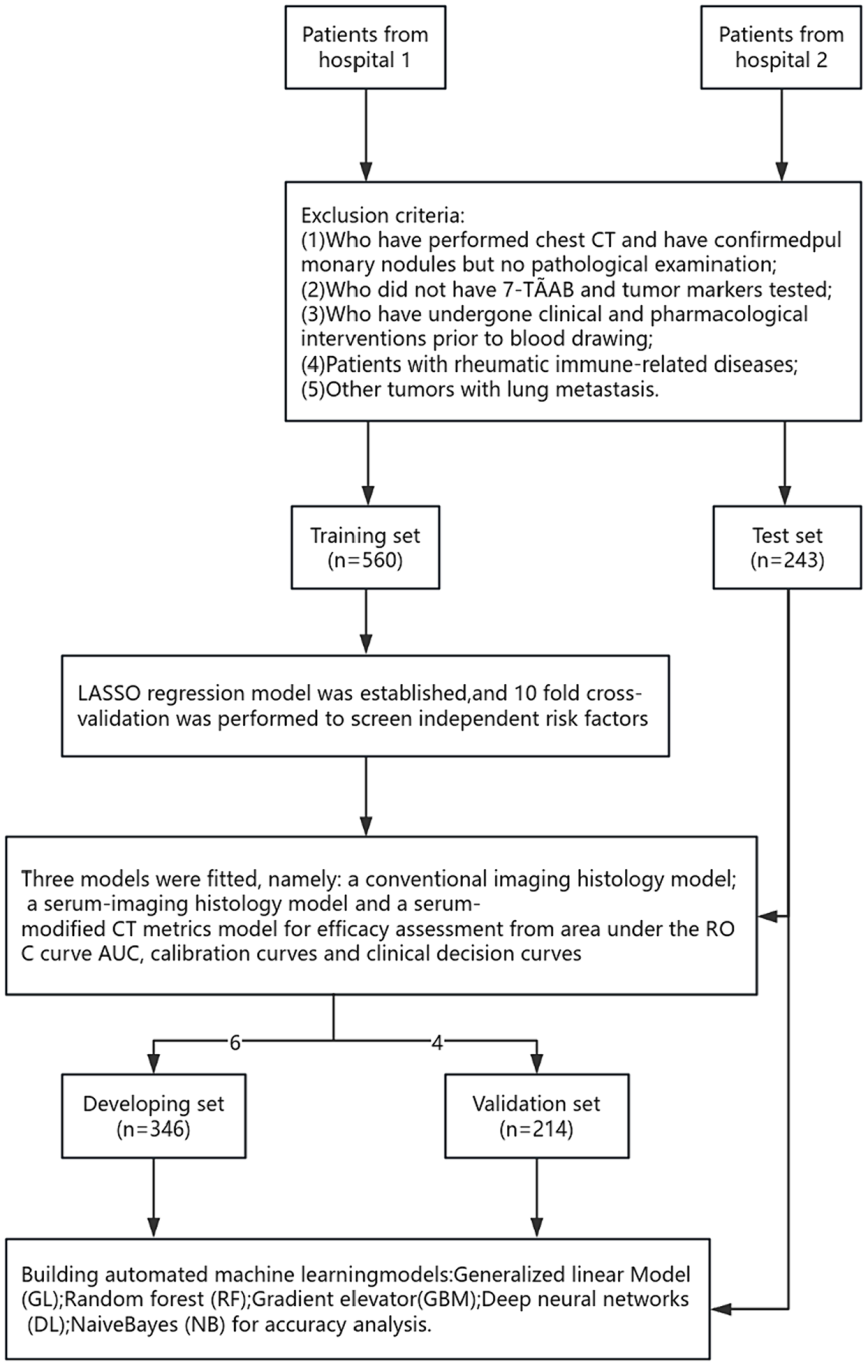
Roadmap for the research program.

A total of 803 lung cancer patients were included in this study, with 376 cases (47.0%) exhibiting infiltrative lesions. The study protocol is detailed in Figure 1. Among them, 560 patients from the Affiliated Changshu Hospital of Soochow University (Hospital 1) were included in the training set. Nantong Eighth People’s Hospital (Hospital 2) contributed 243 patients as the testing set. In the training set, 64.3% (360/560) were male and 35.7% (200/560) were female, with a median age of 55 years. In the testing set, females were more common in the infiltrative group, and the age range of 40–60 years was the peak incidence, consistent with previous reports (16). There were no statistically significant differences between the two groups in terms of age, CY211, NSE, and Leafing (*p* > 0.05). Details are shown in Table 1.

**Table 1:** Baseline characteristics of patients in training and test groups.

Variable	Group	Training data set (*n* = 560)		Z/χ^2^	*p*	Test data set (*n* = 243)		Z/χ^2^	*p*
		Non-infiltration (*n* = 297)	Infiltrate (*n* = 263)			Non-infiltration (*n* = 130)	Infiltrate (*n* = 113)		
Age	–	53.8 (46.4, 64.57)	54.4 (46.32, 62.45)	−0.593	0.553	54.75 (43.25, 64.24)	55.09 (47.86, 62.49)	−0.478	0.633
Gender	男	174 (58.6%)	186 (70.7%)	8.949	0.003	55 (42.3%)	38 (33.6%)	1.928	0.165
	女	123 (41.4%)	77 (29.3%)			75 (57.7%)	75 (66.4%)		
VEGF	–	128.53 (82.68, 172.74)	152.29 (92.31, 214.55)	−3.583	<0.001	117.47 (70.11, 159.96)	153.05 (106.53, 210.52)	−3.833	<0.001
CEA	–	2.15 (1.62, 2.73)	2.56 (1.79, 3.54)	−4.926	<0.001	2.79 (1.98, 3.61)	2.00 (1.46, 2.70)	−4.979	<0.001
CY211	–	2.02 (1.48, 2.67)	1.90 (1.48, 2.34)	−1.896	0.058	1.86 (1.53, 2.46)	2.09 (1.51, 2.56)	−1.447	0.148
NSE	–	13.12 (11.56, 14.61)	13.37 (11.39, 15.49)	−1.029	0.303	13.21 (11.03, 15.55)	13.44 (12.05, 14.98)	−0.540	0.589
SCC	–	0.71 (0.53, 0.87)	0.87 (0.56, 1.20)	−5.389	<0.001	0.90 (0.61, 1.25)	13.44 (12.05, 14.98)	−4.263	<0.000
proGRP	–	41.91 (30.01, 53.17)	37.06 (29.09, 44.52)	−3.686	<0.001	38.22 (30.20, 46.68)	43.94 (29.60, 55.10)	−2.362	0.018
Malignant.probability	–	50.36 (34.13, 68.29)	57.73 (41.70, 72.57)	−5.325	<0.001	45.5 (30.75, 70.0)	61.0 (44.5, 74.5)	−3.165	0.002
Mean.CT.value	–	−255.48 (−395.77, −99.97)	−462.50 (−571.97, −361.17)	−12.247	<0.001	−255.09 (−392.49, −72.21)	−460.62 (−579.04, −345.02)	−7.952	<0.001
Nodule.diameter	–	9.0 (6.0, 12.0)	21.0 (13.0, 25.0)	−13.443	<0.001	9.0 (6.0, 12.0)	20.0 (14.0, 23.0)	−9.887	<0.001
Proportion.of.solidity	–	0.5 (0.3, 0.7)	0.6 (0.4, 0.8)	−4.649	<0.001	0.495 (0.29, 0.68)	0.61 (0.41, 0.77)	−2.839	0.005
TAABs	NO	266 (89.6%)	193 (73.4%)	24.696	<0.001	111 (85.4%)	88 (77.9%)	2.298	0.130
	YES	31 (10.4%)	70 (26.6%)			19 (14.6%)	25 (22.1%)		
Vacuolar	NO	246 (82.8%)	218 (82.9%)	0.000	0.985	105 (80.8%)	74 (65.5%)	7.278	0.007
	YES	51 (17.2%)	45 (17.1%)			25 (19.2%)	39 (34.5%)		
Burr	NO	180 (60.6%)	173 (65.8%)	1.602	0.206	77 (59.2%)	82 (72.6%)	4.753	0.029
	YES	117 (39.4%)	90 (34.2%)			53 (40.8%)	31 (27.4%)		
Leafing	NO	264 (88.9%)	238 (90.5%)	0.387	0.534	123 (94.6%)	109 (96.5%)	0.476	0.490
	YES	33 (11.1%)	25 (9.5%)			7 (5.4%)	4 (3.5%)		
BV	NO	108 (36.4%)	98 (37.3%)	0.048	0.826	53 (40.8%)	30 (26.5%)	5.436	0.020
	YES	189 (63.6%)	165 (62.7%)			77 (59.2%)	83 (73.5%)		
PI	NO	189 (63.6%)	140 (53.2%)	6.231	0.013	79 (60.8%)	77 (68.1%)	1.430	0.232
	YES	108 (36.4%)	123 (46.8%)			51 (39.2%)	36 (31.9%)		
AB	NO	271 (91.2%)	242 (92.0%)	0.107	0.743	116 (89.2%)	110 (97.3%)	6.118	0.013
	YES	26 (8.8%)	21 (8.0%)			14 (10.8%)	3 (2.7%)		

VEGF indicates vascular endothelial growth factor; CEA, carcinoembrionic antigen; CY211, cytokeratin fragment 19; NSE, neuron specific enolase; SCC, squamous cell carcinoma antigen; ProGRP, progastrin releasing peptide; 7-TAAB, seven tumor-associated autoantibodies; Vacuolar, vacuole sign; Burr, spicule sign; Leafing, lobulation; BV, pulmonary nodular vascular passage; PI, pleural indentation; AB, air bronchogram.

### 3.2 Model construction and predictive performance comparison

#### 3.2.1 LASSO regression feature screening and LR model construction

**Figure 2:**
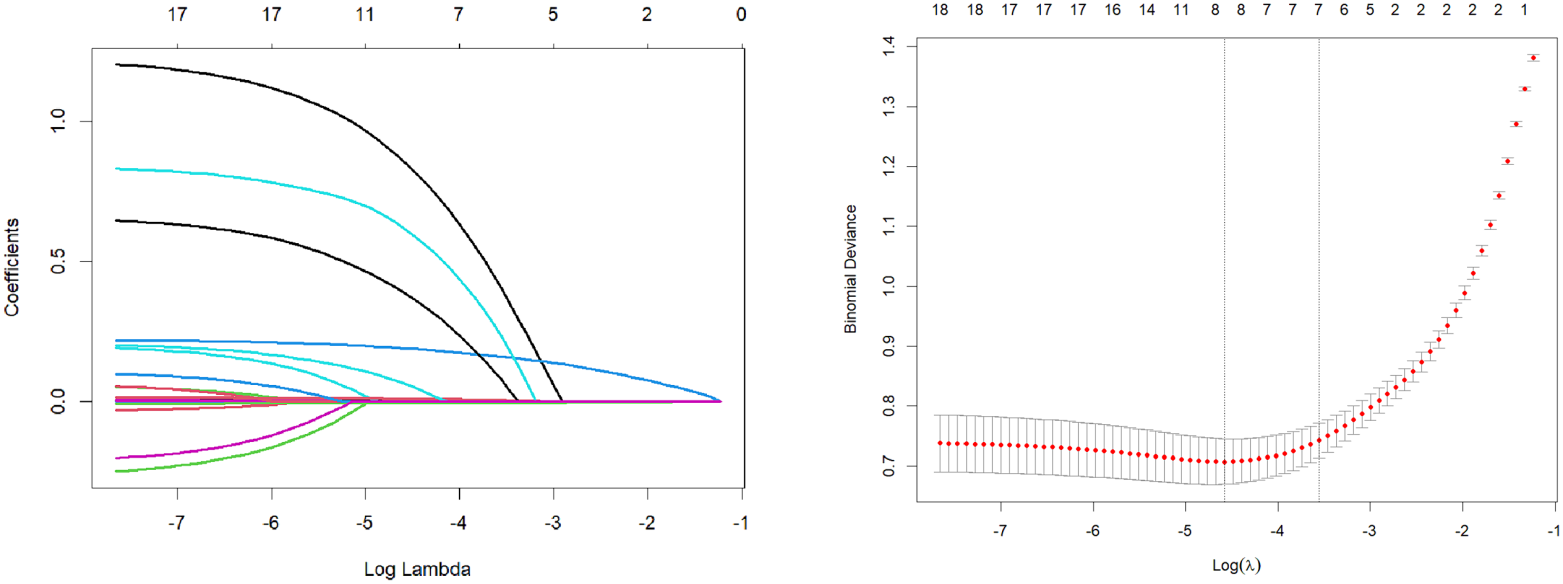
Lasso regression variable screening.

Considering the potential issue of multicollinearity among variables, we employed the LASSO regression model with the introduction of the L1 regularization coefficient. Through 10-fold cross-validation, we obtained the minimum standard lambda and selected 8 variables as independent risk factors from 19 variables. These variables included VEGF, TAABs, malignancy probability, average CT value, nodule diameter, solid proportion, gender, and pleural retraction, as shown in Figure 2.

**Figure 3:**
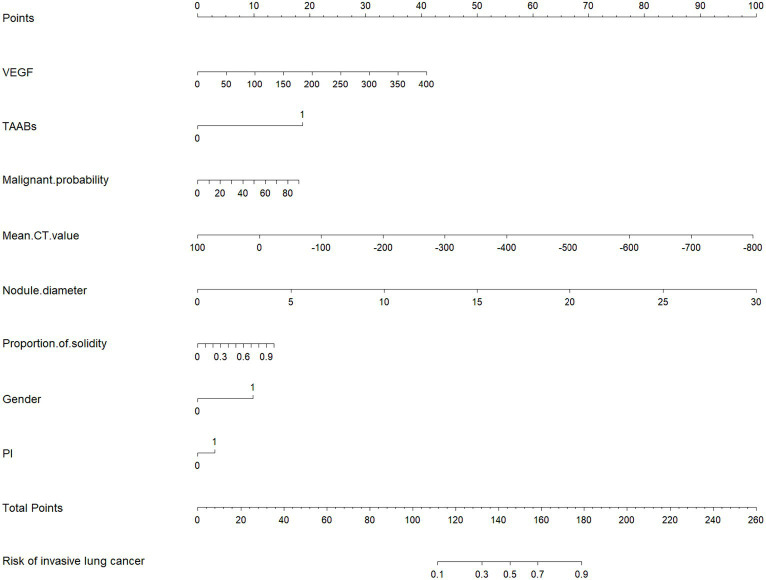
Nomogram (column line graph).

The selected features were fitted to construct a serum-modified CT index model, and a Nomogram plot was generated to score the features (see Figure 3). The total score obtained by summing the scores of each feature allows estimation of the probability of developing infiltrative lesions in lung cancer. The study showed that when the total score of the Nomogram for lung cancer infiltrative lesions exceeds 180, the risk of lesions is over 90%.

**Figure 4:**
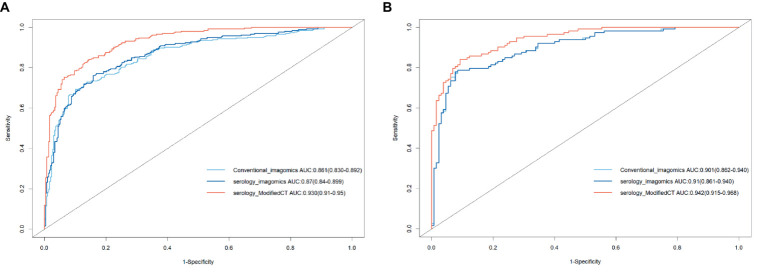
Three model ROCs; **(A)** Training set; **(B)** Testing set.

**Figure 5:**
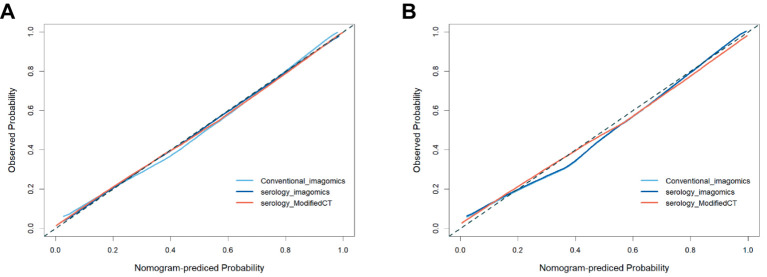
Three model calibration curves; **(A)** Training set; **(B)** Test set.

**Figure 6:**
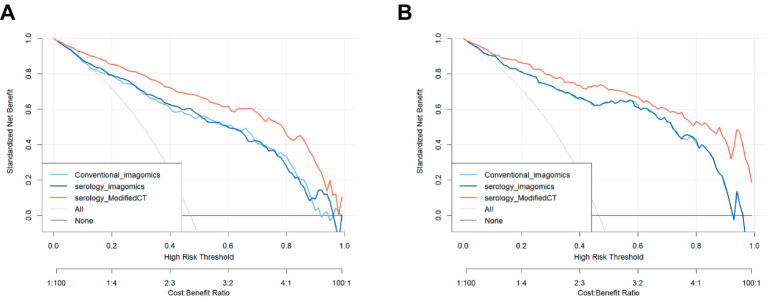
DCA curves for the three models; **(A)** Training set; **(B)** Test set.

To further analyze the stability and clinical utility of the serum-modified CT index model, we compared the serum-modified CT index model with conventional imaging omics models and serum-imaging omics models in both the training and testing sets using ROC curve analysis, clinical calibration curve, and clinical decision curve analysis (DCA). The conventional imaging omics model consisted of nodule diameter, solid proportion, gender, and pleural retraction. The serum-imaging omics model included VEGF, TAABs, nodule diameter, solid proportion, gender, and pleural retraction. The serum-modified CT index model comprised VEGF, TAABs, malignancy probability, average CT value, nodule diameter, solid proportion, gender, and pleural retraction. In the training set, the ROC curve analysis revealed that the areas under the curve (AUC) for the conventional imaging omics model, serum-imaging omics model, and serum-modified CT index model were 0.861, 0.87, and 0.930, respectively (see Figure 4A). In the testing set, the AUC values were 0.901, 0.91, and 0.942 for the conventional imaging omics model, serum-imaging omics model, and serum-modified CT index model, respectively (see Figure 4B). The calibration curves for the training and testing sets (see Figures 5A,B) demonstrated that the estimated risks of the serum-modified CT index model were very close to the actual risks, indicating high reliability. The clinical decision curve analysis (DCA) showed that, across most threshold ranges, the net benefit of the serum-imaging omics model was greater than that of the conventional imaging omics model and serum-imaging omics model in both the training and testing sets, with the serum-imaging omics model outperforming the conventional imaging omics model (see Figures 6A,B).

#### 3.2.2 Machine learning model construction and performance comparison

Using the H2OAutoML platform, automatic training and adjustment of models were conducted within a 5 min time limit, resulting in the construction of 75 models. However, due to limited interpretability and the presence of stacked ensemble models, these models were simplified, and the main algorithms involved were extracted, including Generalized Linear Model (GLM), Random Forest (RF), Gradient Boosting Machine (GBM), Deep Neural Network (DL), and Naive Bayes (NB). Among these models, the GBM model outperformed others, achieving the highest values for AUC, accuracy, and F1-Measure on both validation and testing sets, and hence was considered the optimal model. As shown in Table 2, on the validation and testing sets, the AUC values obtained by the GBM algorithm were higher than those obtained by GLM, RF, DL, and NB algorithms, with values of (0.931, 0.99) compared to (0.917, 0.942), (0.918, 0.986), (0.901, 0.948), and (0.908, 0.944), respectively. Furthermore, compared to GLM, RF, DL, and NB algorithms, the GBM algorithm also achieved the highest accuracy, with values of (0.857, 0.955), (0.854, 0.864), (0.838, 0.947), (0.819, 0.877, 0.844, 0.889), respectively. Among these models, the RF model exhibited the highest sensitivity in both the validation and testing sets, with values of 0.914 and 0.991, respectively. Both RF and GLM models demonstrated good performance in terms of AUC, sensitivity, specificity, and accuracy.

**Table 2:** Comparison of AutoML model performance in predicting lung cancer infiltration in the test cohort.

Targets	GLM		RF		GBM		DL		NB	
	Validation	Test set	Validation	Test set	Validation	Test set	Validation	Test set	Validation	Test set
Accuracy	0.854	0.864	0.838	0.947	0.857	0.955	0.819	0.877	0.844	0.889
AUC	0.917	0.942	0.918	0.986	0.931	0.99	0.901	0.948	0.908	0.944
Sensitivity	0.771	0.903	0.914	0.991	0.893	0.982	0.800	0.885	0.843	0.885
Specificity	0.917	0.831	0.779	0.908	0.829	0.931	0.834	0.869	0.845	0.892
PPV	0.878	0.823	0.762	0.903	0.801	0.925	0.789	0.855	0.808	0.877
NPV	0.838	0.908	0.922	0.992	0.909	0.984	0.844	0.897	0.874	0.899
LR+	9.309	5.334	4.137	10.737	5.213	14.189	4.827	6.767	5.448	8.217
LR−	0.249	0.117	0.110	0.010	0.129	0.019	0.240	0.132	0.186	0.129
F1-Measure	0.821	0.861	0.831	0.945	0.845	0.953	0.794	0.870	0.825	0.881

AUC indicates area under the curve; PPV, positive predictive value; NP, negative predictive value; LR−, negative likelihood ratio; LR+, positive likelihood ratio; GLM, Generalized linear model; RF, Random forest; GBM, gradient boosting machine; DL, deep neural net; NB, Naive Bayes.

### 3.3 Overall feature interpretability analysis

**Figure 7:**
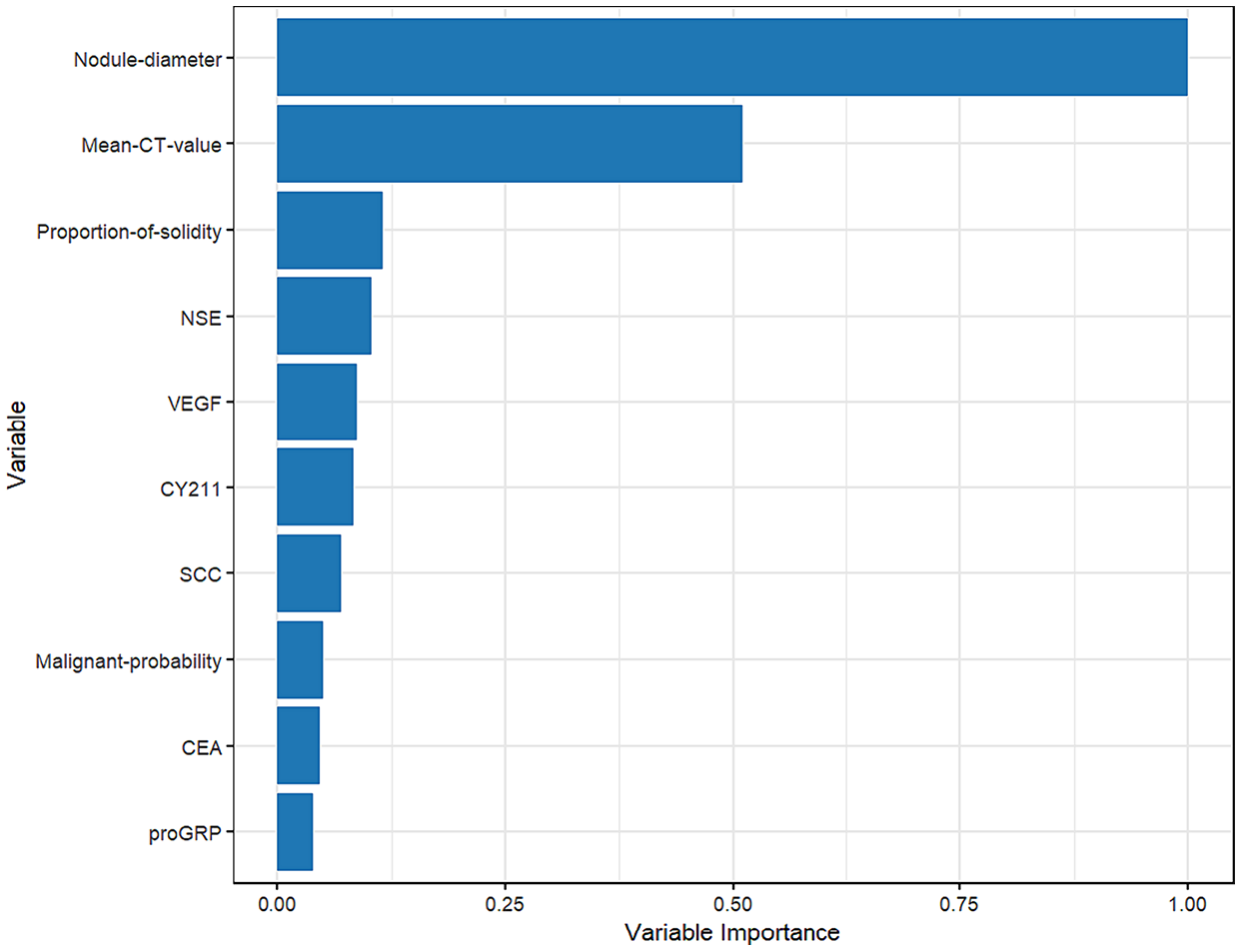
Plot of the importance ranking of the GBM model variables in the test set.

Figure 7 shows that nodule diameter size is the most important feature, followed by average CT value, solid proportion, NSE, VEGF, CYFRA21-1, SCC, malignancy probability, CEA, and proGRP. Additionally, nodule diameter size, average CT value, malignancy probability, solid proportion, and VEGF were identified as important feature variables shared by both the GBM and logistic regression models.

**Figure 8:**
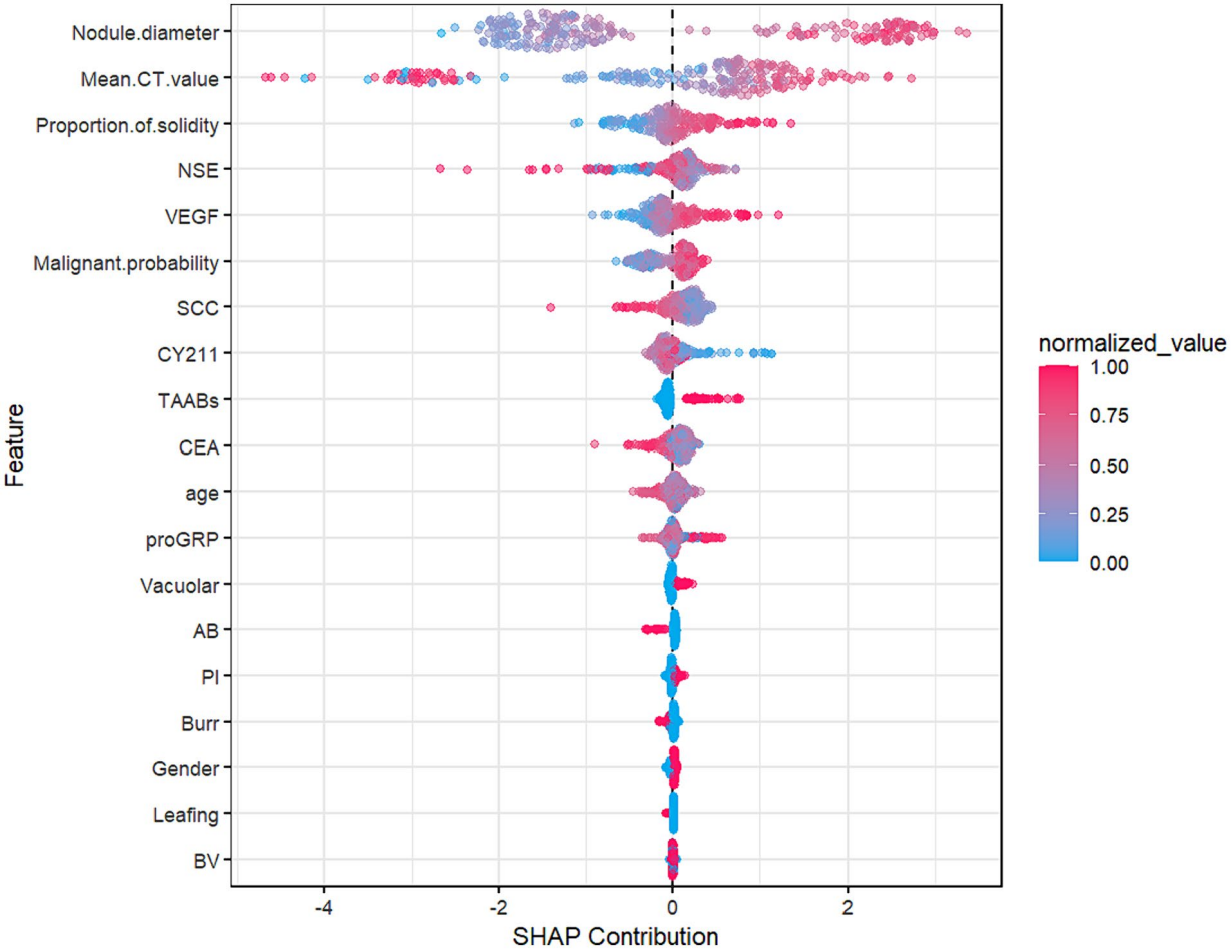
Summary plot of GBM model SHAP in the test set.

Figure 8, the SHAP summary plot, displays the impact of all features on the predictive performance of the GBM model in the testing set. The x-axis represents the SHAP values, indicating the contribution of features to the overall prediction. A SHAP value greater than 0 indicates a positive contribution, meaning that as the variable’s value approaches 1, the likelihood of infiltration in patients increases. For example, on the SHAP plot corresponding to nodule diameter, red points are mainly located to the right of the zero axis, while blue points are more on the left, suggesting that as the nodule diameter increases, the likelihood of infiltrative lesions in lung nodules also increases.

### 3.4 Individual feature interpretability analysis

**Figure 9:**
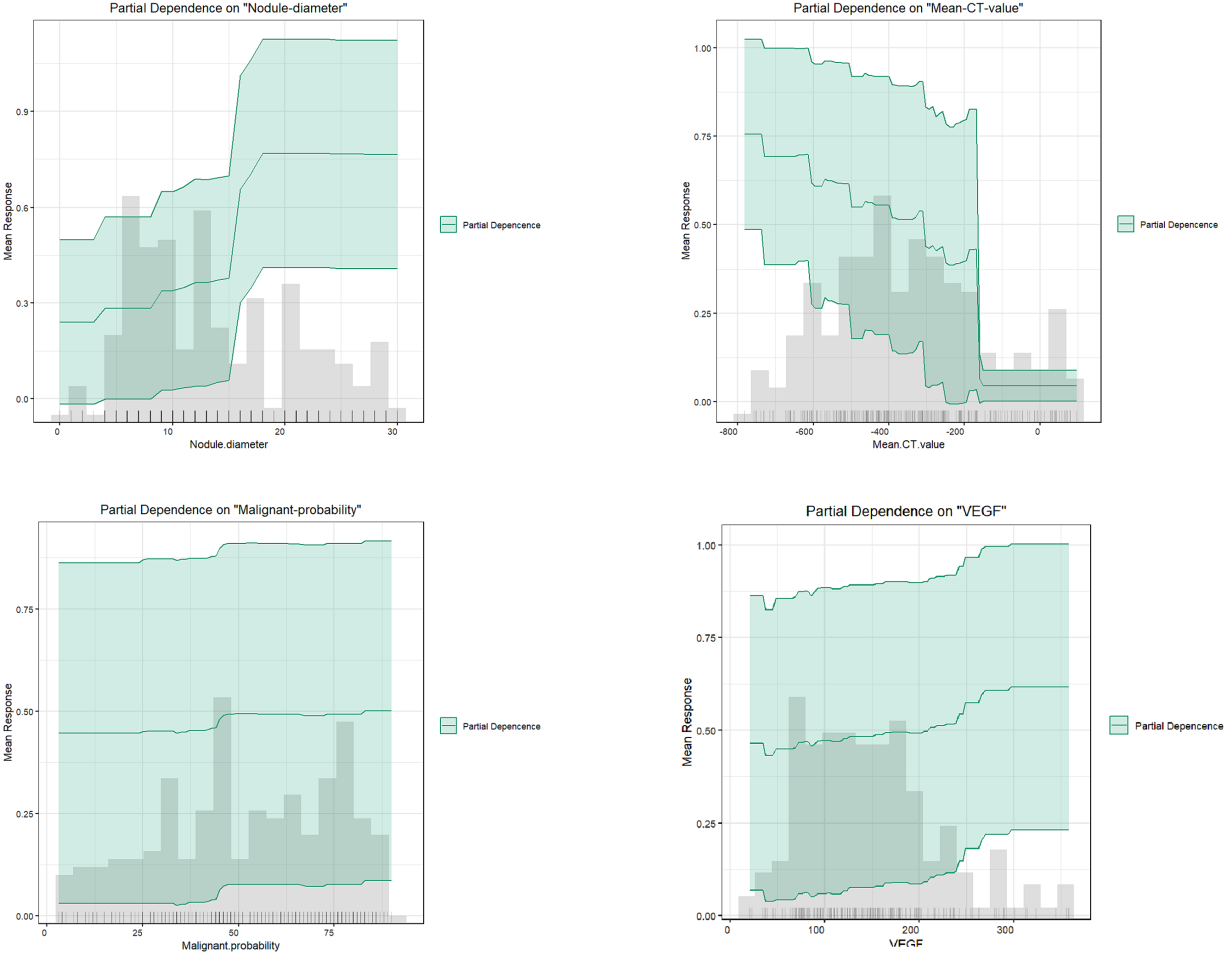
Partial dependence plots.

As shown in Figure 9, partial dependence plots illustrate the impact of individual features on the final discrimination of the GBM model and their distribution in the dataset. Nodule diameter size, malignancy probability, and VEGF are positively correlated with the likelihood of infiltrative lesions. Nodule diameter is mainly distributed below 15 mm, but for lung cancer patients falling between 15 and 18 mm, there is a higher likelihood of infiltrative lesions, necessitating regular follow-up. As the average CT value gradually increases, it tends to indicate non-invasive lung cancer, particularly in patients with values above −200, essentially ruling out the possibility of infiltrative lung cancer.

**Figure 10:**
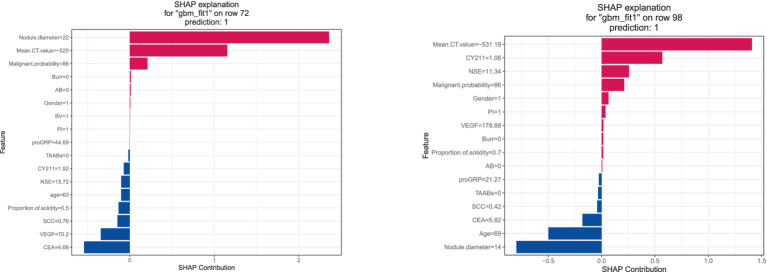
SHAP interpretation diagram.

The SHAP explanation illustrates the feature contributions for specific instances. As depicted in Figure 10, for instance 72, with a nodule diameter of 22 mm, average CT value of -525HU, and malignancy probability of 86%, these factors significantly contribute to the model’s final determination of infiltrative lung cancer. Conversely, in instance 98, although the nodule diameter is below 15 mm, predictions of infiltrative lung cancer are made based on factors such as average CT value, NSE value, and malignancy probability.

## 4 Discussion

Lung cancer ranks among the most prevalent and fatal malignancies globally, with adenocarcinoma being the most common histological subtype. Accurate differentiation between non-invasive and invasive lung cancer significantly impacts patient prognosis and survival. Therefore, constructing early lung cancer infiltration risk prediction models is crucial. In recent years, many researchers have built clinical risk prediction models for early lung cancer patients using multivariable logistic regression and selected feature variables such as low-dose CT (LDCT), seven autoantibodies, and other biomarkers (17–21). Unlike many previous studies, this research incorporates AI-improved malignancy probability and average CT value into the category of risk factors and compares models constructed by traditional LR regression with those built by AutoML algorithms to assess their efficacy and accuracy.

Feature interpretability analysis results show that the most crucial feature of the GBM model is nodule diameter size, consistent with the results of the logistic regression model in this study and the risk factors for lung nodule benignity/malignancy reported in related studies (22, 23). Other researchers have pointed out that as nodule diameter increases, the likelihood of malignancy also increases. For instance, nodules below 5 mm have a malignancy rate of only 1%, while those between 5 and 10 mm have a malignancy rate of 25% (24). In this study, we found that nodules larger than 15 mm have a higher malignancy probability, particularly between 15 and 18 mm, where infiltration is more likely to occur. Therefore, patients should have shorter follow-up intervals, and clinicians should pay close attention to patients with nodules larger than 15 mm, increasing the frequency of follow-up visits. This finding is consistent with other research (25, 26).

With the development and application of artificial intelligence technology, AI-based medical imaging has been widely used in clinical diagnosis and treatment, particularly in lung cancer early screening, significantly improving lung nodule detection rates and reducing the rate of missed small lesions. This study demonstrates that AI-enhanced CT indices significantly contribute to the discrimination of infiltrative lung cancer, enhancing lesion identification accuracy. However, there are limitations. According to previous studies, although CT AI has higher positive predictive values and sensitivity, its specificity is not ideal, ranging from 70 to 80% (27–30). Therefore, relying solely on radiological imaging to differentiate between benign and malignant lung nodules is too one-sided. This study established a predictive model combining AI with other laboratory indicators to improve the specificity and accuracy of lung nodule detection.

In recent years, laboratory indicators for lung cancer have mainly focused on primary lung cancer biomarkers and seven lung cancer autoantibodies. In contrast to artificial intelligence CT, these indicators have high specificity but low sensitivity when used alone. Therefore, they are typically used in combination for early lung cancer screening. Vascular endothelial growth factor (VEGF) levels serve as an independent risk factor for lung cancer infiltration, as evidenced by significant expression in both LR and GBM models. Studies have shown that VEGF can increase vascular permeability (31–33), thereby promoting tumor metastasis, and its overexpression indicates poor prognosis in lung cancer. Therefore, patients with abnormal VEGF levels should be closely monitored, and further diagnostic and clinical intervention measures should be implemented. Detection of serum lung cancer autoantibodies has a certain clinical decision-making value for lung cancer diagnosis (34–36), although in this study there was a statistically significant difference between the non-infiltrating group and the infiltrating group in the training set, but showed no statistically significant difference between the non-infiltrating group and the infiltrating group in the test set, which indicates that the 7-item serum lung cancer autoantibody test is not suitable to be applied alone in discriminating non-infiltrating versus infiltrating early stage lung cancer, and that it needs to be combined with other indicators for prediction.

In addition, we used five different ML algorithms to construct a high-precision prediction model. The GBM model showed optimal prediction efficacy on both the test and validation sets and achieved higher AUC and accuracy than the LDCT+7-TABBs model constructed by Zhong et al. (37), which fully demonstrated that the CT metrics modified by AI are more accurate, and can provide more comprehensive and high-quality information for clinically assisted diagnosis and treatment. By accurately predicting the invasiveness of early lung nodules, this study can help patients receive earlier treatment, thereby improving survival rates and prognosis. The blind validation using a validation set and external dataset with larger sample sizes and higher external validity mitigated potential biases arising from unique circumstances at a single research center. However, our study also has some limitations. Firstly, it only studied benign and infiltrative lung cancer categories, necessitating the expansion of case numbers to further classify lung cancer. Additionally, this study is retrospective, which introduces selection bias, highlighting the need for more prospective studies for external validation.

## 5 Conclusion

A predictive early-stage lung cancer infiltrative machine learning model was constructed and compared by combining improved CT indices with serological markers, using SHAP to elucidate the clinical significance of each risk factor in predicting infiltrative lesions in early-stage lung cancer patients. The CT indices improved by artificial intelligence are closely associated with lung cancer infiltrative features, holding significant application value in future clinical research. This combination can assist clinicians in implementing early clinical interventions, providing more comprehensive information for self-screening and disease management of early-stage lung cancer patients, thereby preventing and reducing the risk of infiltration.

## Data availability statement

The raw data supporting the conclusions of this article will be made available by the authors, without undue reservation.

## Ethics statement

The studies involving humans were approved by Ethics Management Committee of Changshu No.1 People’s Hospital. The studies were conducted in accordance with the local legislation and institutional requirements. Written informed consent for participation was not required from the participants or the participants’ legal guardians/next of kin in accordance with the national legislation and institutional requirements.

## Author contributions

LM: Writing – original draft. PZ: Data curation, Visualization, Writing – review & editing. KX: Methodology, Writing – review & editing.

## Footnotes

^1^ www.h2o.ai
